# A novel in-situ method to determine the respiratory tract deposition of carbonaceous particles reveals dangers of public commuting in highly polluted megacity

**DOI:** 10.1186/s12989-022-00501-x

**Published:** 2022-09-15

**Authors:** Leizel Madueño, Simonas Kecorius, Jakob Löndahl, Jürgen Schnelle-Kreis, Alfred Wiedensohler, Mira Pöhlker

**Affiliations:** 1 Experimental Aerosol and Cloud Mircophysics, Leibniz-Institute for Tropospheric Research, Leipzig, Germany; 2 Ergonomics and Aerosol Technology, Lund University, Lund, Sweden; 3 Comprehensive Molecular Analytics, Helmholtz Zentrum München—German Research Center for Environmental Health, München, Germany; 4 World Calibration Center for Aerosol Physics, Leipzig, Germany; 5 Present Address: Institute of Epidemiology, Helmholtz Zentrum München—German Research Center for Environmental Health, Ingolstädter Landstr. 1, 85764 Neuherberg, Germany

**Keywords:** Lung deposition, Air pollution, Health effects, Toxicity, Black carbon, Traffic-related particulates, Transport microenvironment

## Abstract

**Background:**

Exposure to air pollutants is one of the major environmental health risks faced by populations globally. Information about inhaled particle deposition dose is crucial in establishing the dose–response function for assessing health-related effects due to exposure to air pollution.

**Objective:**

This study aims to quantify the respiratory tract deposition (RTD) of equivalent black carbon (BC) particles in healthy young adults during a real-world commuting scenario, analyze factors affecting RTD of BC, and provide key parameters for the assessment of RTD.

**Methods:**

A novel in situ method was applied to experimentally determine the RTD of BC particles among subjects in the highly polluted megacity of Metro Manila, Philippines. Exposure measurements were made for 40 volunteers during public transport and walking.

**Results:**

The observed BC exposure concentration was up to 17-times higher than in developed regions. The deposition dose rate (DDR) of BC was up to 3 times higher during commute inside a public transport compared to walking (11.6 versus 4.4 μg hr^−1^, respectively). This is twice higher than reported in similar studies. The average BC mass deposition fraction (DF) was found to be 43 ± 16%, which can in large be described by individual factors and does not depend on gender.

**Conclusions:**

Commuting by open-sided public transport, commonly used in developing regions, poses a significant health risk due to acquiring extremely high doses of carcinogenic traffic-related pollutants. There is an urgent need to drastically update air pollution mitigation strategies for reduction of dangerously high emissions of BC in urban setting in developing regions. The presented mobile measurement set-up to determine respiratory tract deposition dose is a practical and cost-effective tool that can be used to investigate respiratory deposition in challenging environments.

**Supplementary Information:**

The online version contains supplementary material available at 10.1186/s12989-022-00501-x.

## Background

Exposure to air pollution has been recognized as a significant contributor to the global burden of disease [1], causing three [2] to nine [3] million deaths worldwide. To investigate the association of exposure to air pollution with adverse health effects, researchers have long relied on particulate matter (PM) with an aerodynamic diameter of less than 2.5 μm (PM_2.5_) [4]. However, more and more pieces of evidence arise indicating that individual components of PM_2.5_ may have unequal health effects [5, 6]. Additional segregation between PM constituents may help better understand the health impacts of air pollution [7]. The International Agency for Research on Cancer (IARC) has listed fine dust, i.e., traffic-related particulates, as carcinogenic to humans (IARC Group 1) because of its high content of heavy metals and sulfur oxides [8], causing a particularly negative effect on human health [9]. Several studies proposed that black carbon, a major component of traffic-related emissions, may act as a carrier of carcinogenic compounds [10, 11]. However, black carbon exposure has been studied less than PM, therefore, their real effects are not yet established and are not included in the estimation of mortality rates.

Exposure can be quantified as a time-weighted concentration experienced by a person in a microenvironment, which is a function of the frequency, intensity, and duration of contact with a pollutant [12]. A large fraction (up to 30%) of air pollution exposure to black carbon is experienced during transportation or commuting [13]. Transport microenvironments (TMEs) such as walking, cycling, car, bus, and open-sided vehicles, are the most common modes of local transportation. Most of the existing review articles that assess pollutant exposures in TMEs are conducted in developed regions (e.g., Europe [14]). In low-to-middle income regions (LMIR) pollution exposure assessments remain limited. Because of the different vehicle fleet compositions, road configurations, and driving behavior in LMIR, the TME exposure studies conducted elsewhere may not be applicable.

To better understand the effects of air pollution exposure on human health, it is crucial to determine how much of the inhaled pollutants that are deposited in the respiratory tract. A common practice for assessing the respiratory tract deposition (RTD) of pollutants is to either apply numerical (in silico) or semi-empirical models (e.g., International Commission of Radiological Protection [15] and Multiple-Path Particle Dosimetry models [16]) or to perform laboratory and real-life in situ experiments involving human subject (e.g., RESPI [17], MERDOC [18], single breath measurement [19]). Additionally, other prior approaches (ab initio) [20] were used in some studies, however, the assumptions made (i.e., all inhaled particles are deposited) make such methods more susceptible to large uncertainties. Choosing one of the methods to estimate RTD depends on application and requirements, and while each method poses its advantages and disadvantages, in situ measurements arguably have the least assumptions among other available methods. In experiments involving humans, the complexity of the respiratory system, which depends on various conditions, can be fully represented. With that being said, in situ-based RTD assessments are usually more complicated to execute due to the involvement of human subjects, the requisite for ethical approval, and often an expensive and complex experimental setup. This may be the reason for only limited RTD studies in LMIR. Without in situ measurements, important parameters, e.g., particle uptake kinetics and personal breathing pattern, which are dependent on physiology and ethnicity, remain unknown, which may increase the uncertainties in the assessment of deposition dose.

A review of in situ RTD studies over the past years (Additional file 1: Tables S1–S2) shows that most of the studies were performed in laboratory setups with predefined exposure concentrations [21, 22], aerosol sources [17, 19, 21–24], and were mostly limited to passive activity, e.g., sitting [22, 23, 25–27]. Most importantly, no previously conducted studies were designed to reflect a real-world exposure scenario, i.e., RTD in a microenvironment during actual daily activity. Due to the complexity of aerosol in situ RTD studies, the majority of published experiments were done on a limited number of study participants (n < 10). It becomes evident that the information about RTD studies in real-world scenarios remains scarce. This is especially critical for developing regions, where high exposure concentrations and air quality continually worsens [28]. As highlighted in the recent update of air quality guidelines by the World Health Organization (WHO) [28], it is vital to assess the shape of exposure–response relationships at both low and high air pollution levels. Assessing the real exposure and real lung deposition of pollutants will help in establishing exposure profiles, especially to represent the complex LMIR environments. This study serves as a step towards understanding the health effects of exposure to extremely high concentrations of carbonaceous particles in outdoor air in developing regions.

To improve the understanding of RTD in highly polluted environments, we deployed our previously developed method (MERDOC [18]) focusing on the respiratory deposition of equivalent black carbon (BC) particles in different TMEs. Metro Manila, the capital of the Philippines, was chosen as the study domain, representing LMIR in Southeast Asia. Metro Manila is a highly polluted urban environment [29, 30], with main air constituents, e.g., ultrafine particles, BC, originating from the most commonly used public utility vehicle (PUV, *Jeepney*) [29], which is powered by old-technology diesel engines. High exposure concentrations, combined with elongated commuting times [31]are expected to pose a significant health risk that many developing megacities have not yet assessed.

The aims of this study are to 1) quantify RTD of BC mass in healthy young adults during a real-world commuting scenario and 2) analyze factors (including both physical and environmental) affecting RTD of BC. The results from this study provide insights into the respiratory tract deposition of carbonaceous particles in different TMEs. Above all, this study raises the awareness of air pollution in developing regions, provides information on the physical respiratory parameters of young adults, and provides data to assess health-related effects due to exposure to high concentrations of BC.

## Methods

### Study design

In this randomized, crossover study, a total of 45 study participants aged between 18 to 27 years were recruited for investigation of RTD of BC particles during their public commute (only 40 were qualified and selected for the data analysis). Exposure scenarios (starting in a clean or polluted environment) were randomly assigned to each subject on predetermined commuting routes choosing from modes of transportation, i.e., riding public transport and walking. A self-administered questionnaire was used to collect basic information, including name, gender, age, and history of cardiovascular or other diseases. All study participants were subjectively healthy and had no reported history of disease that might affect the lung function or the experiment. The subjects were selected according to the following criteria: no reported history of smoking; no physician-diagnosed cardiovascular, pulmonary, neurological, or other chronic diseases; and non-obese. Prior to the study, informed consent was obtained from all subjects.

### Experimental set-up

**Fig. 1:**
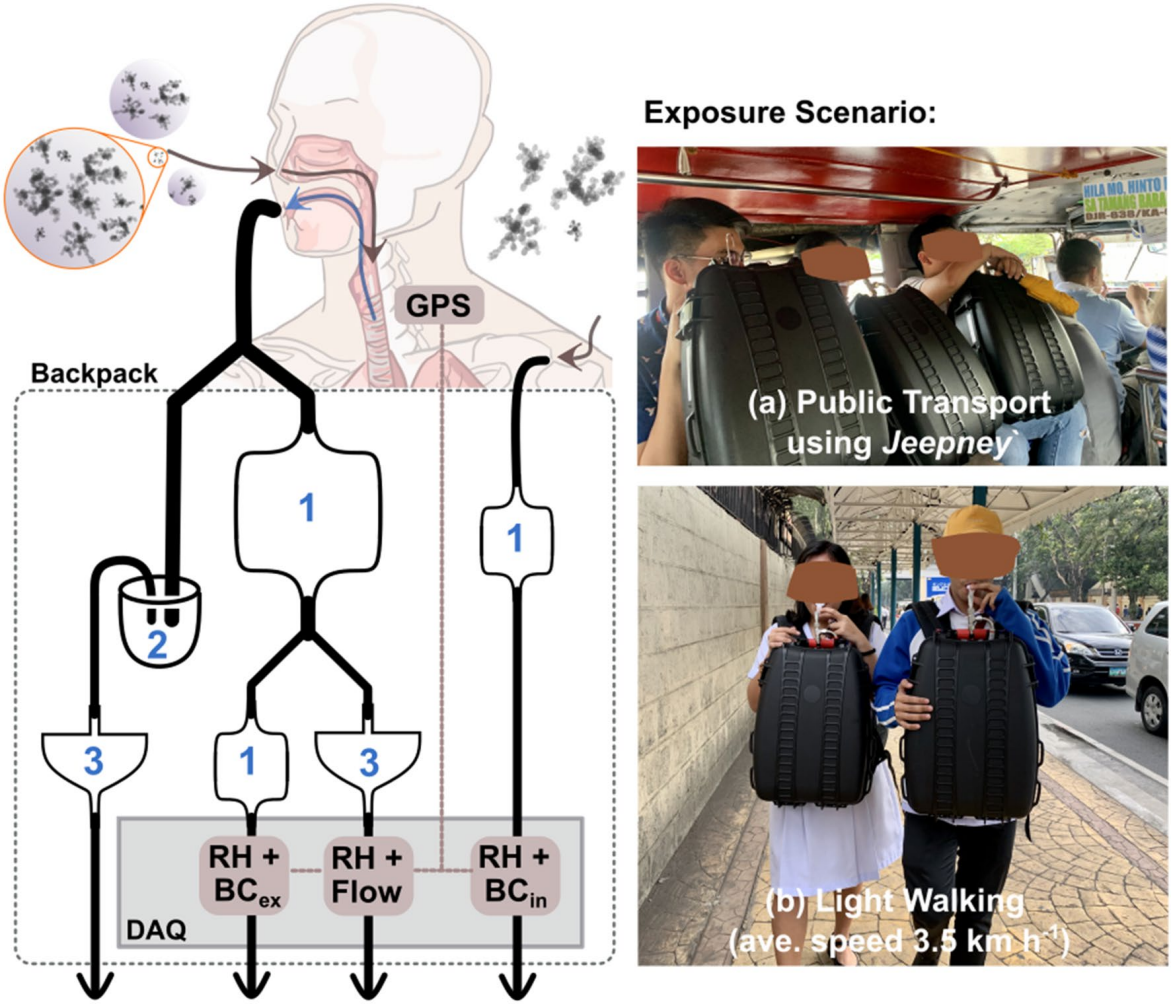
Schematic diagram of the modified MERDOC measurement system. The measurement setup consists of (1) three mixing chambers equipped with silica gel desiccant; (2) exhaled air water trap, and; (3) two high-efficiency particulate filters. A detailed description of the experimental setup is discussed in Madueño et al*.* [18]. Photos on the right show the actual sampling of study participants in different exposure scenarios, **a** public transport and **b** light walking. Please note that the exposure and exhaled BC mass concentrations were measured by separate micro-aethalometers placed in two different backpacks carried by different individuals (study supervisor (BC exposure measurement) and volunteers (exhaled BC mass concentration measurement))

The breathing parameters (calculated from exhaled airflow rate), personal exposure, and respiratory tract deposition of BC were measured using a previously developed measurement system to quantify real-world respiratory tract deposition dose of black carbon (MERDOC). A comprehensive description of the measurement system and data evaluation can be found in Madueño et al. [18]. Several minor changes were made to the previous version of MERDOC (e.g., installation of a bypass for exhaled air, water trap, and double aerosol dryer), which both allowed better aerosol drying and improved system operability without compromising data quality. Briefly, the ambient aerosol is inhaled exclusively through the nose during normal tidal breathing, which is then exhaled through the mouthpiece (i.e., unidirectional flow) into a mixing chamber (filled with silica gel for drying, Fig. 1).

Carbonaceous aerosol particles were sampled from the stream of air at ambient pressure using a micro-aethalometer (model MA200, AethLabs, San Francisco, CA, USA; at a flow rate of 100 ml/min). Exposure concentrations of BC were monitored separately in parallel to exhaled air measurements. Data acquisition (logging BC mass concentrations, relative humidity, and temperature of sampled aerosol, as well as position in 1 s time resolution) was accomplished by 3 separate micro-computers, equipped with MERDOC systems. Two units of mobile MERDOC measurement systems were carried along the predetermined commuting routes by two volunteers (bringing instruments to measure exhaled BC mass concentration) and an experiment supervisor (bringing instruments to measure ambient BC mass concentration). The study participants were instructed on how to properly handle and use the MERDOC measurement system prior to the experiment. Mode of transportation (start and end times; based on position and speed information), exposure and exhaled BC mass concentrations, as well as breathing parameters were determined from recorded MEDOC data in the data evaluation step.

Aerosol particle losses in the instrument for both exhaled air and ambient aerosols, at different airflow rates across the mixing chamber (mimicking real-world exhaled airflow rate), were evaluated in the laboratory prior to the measurement campaign (Additional file 1: Fig. S1). The results from intercomparison between ambient and exhaled BC mass concentration measurement systems are available in Additional file 1: Figs. S2–S3. Additionally, the flow rate of exhaled air through HEPA filter 3 (Fig. 1, bypass line) was evaluated prior to (dry-state), during (moist-state), and after (moist-state) the experiment using Gillian Gilibrator-2 (Sensidyne, St. Petersburg, FL, US) primary standard airflow calibrator (Additional file 1: Fig. S4). No change in flow rate was observed with respect to HEPA filter wetting. The true exhaled airflow rate was calculated from the ratio between supplied and measured flow rates at the system inlet and exhaled air sample line. Laboratory and field evaluations of measurement set-up confirmed inter-system agreement within 10%.

### Exposure procedure

**Fig. 2:**
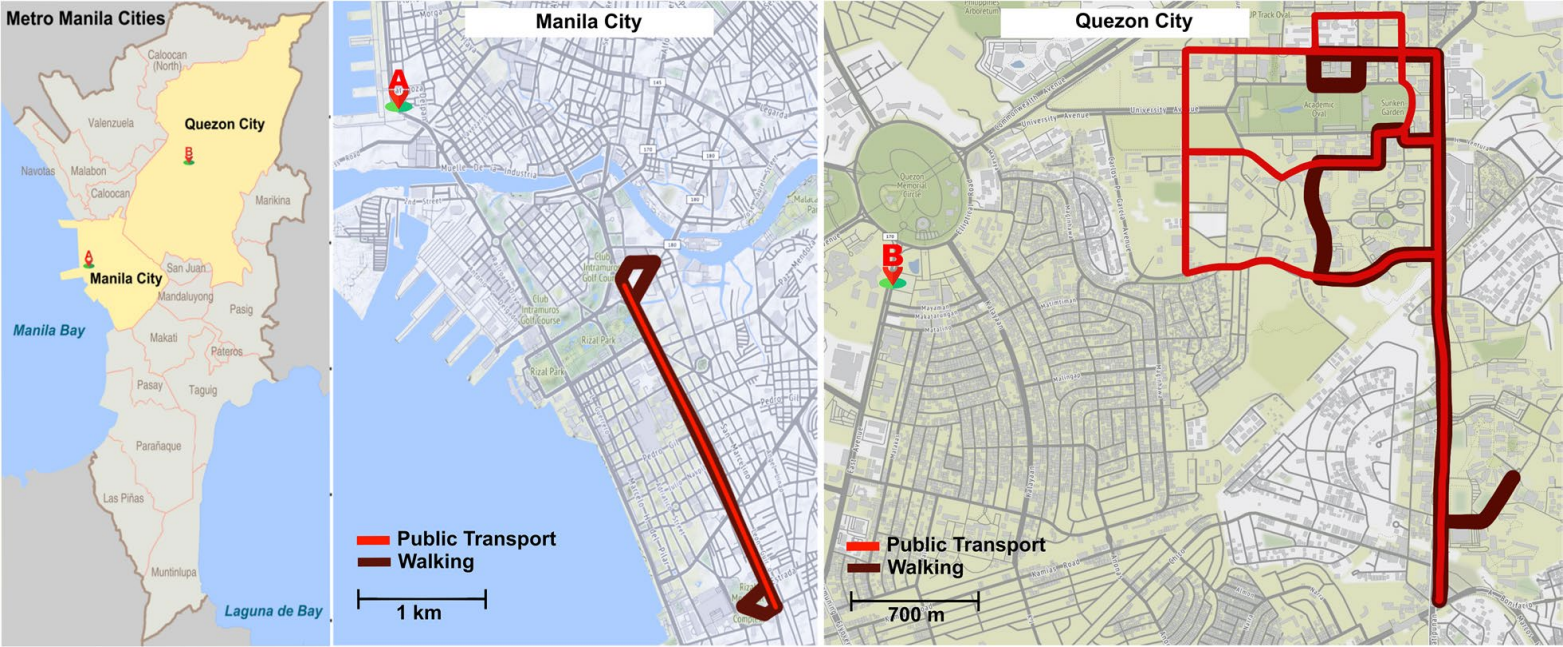
The stationary measurement sites during TAME-BC were located in **A** Manila North Port, Manila City and **B** East Avenue, Quezon City. The exposure of study participants was conducted on a predetermined commuting route within a 5 km radius from the stationary measurement sites. The study participants were given the freedom to choose between commuting by riding *Jeepney* (along the red line) or commuting by walking (along the brown line), or both

The measurement campaign was performed as part of the project “Transdisciplinary Approach to Mitigate Emissions of Black Carbon (TAME-BC) [32]” in Metro Manila, Philippines. The TAME-BC campaign was conducted during the dry season (November 2019–March 2020) in the Philippines. The stationary measurement sites were positioned in the passenger harbor of Manila North Port, Manila City, and on the street side of East Avenue, Quezon City (Fig. 2) to investigate the physical–chemical aerosol properties of a highly urbanized megacity. The exposure measurements presented in this study (from 6 January to 10 March 2020) were carried out within a 5 km radius from the stationary measurement sites. The measurements were conducted on non-rainy days to avoid the washout effects of rainfall on ambient particulate concentration [33] and the convenience of the study participants. During the whole measurement campaign, the daily median temperature and RH were 26 °C (23–31 °C) and 67% (48–82%), respectively.

The study participants were asked to commute in TMEs by (1) public transport; and (2) walking on a curbside (Fig. 1a, b). The commute in public transport was restricted to riding a *Jeepney*, an open-sided vehicle that serves as the cheapest and the most common means of public transportation in the Philippines. The commute by walking on the curbside was instructed to be as casual-paced as possible (average speed of 3.5 km h^−1^). The streets chosen for commuting were significant thoroughfares in Metro Manila with canyon-like topography representing  a polluted road environment. In contrast, a clean environment was characterized by transiting in an open space surrounded by greenery (usually inside a gated university campus).

The study participants traveled on the predefined routes, either in Manila or Quezon City (Fig. 2), between 6 am and 6 pm, with the majority of the time between midday and 4 pm. The participants randomly chose to start commuting either in a polluted or clean environment, with their choice of transportation (e.g., riding public transport and then walking, or vice versa), with the prerequisite to cover both walking and a public transport ride in one trip. A total of 63 periods of walking (723 min) and 97 journeys by public transport (420 min) were made. The commuting routes took at least 30 min to complete. The study supervisor (measuring BC exposure concentration) and participants (measuring exhaled BC mass concentration) traveled closely together (i.e., sat together on public transport and walked closely together) to ensure that the measured ambient concentrations by the supervisor were a suitable estimate of the BC concentrations inhaled by the participants. Before each measurement session, the volunteers were given approx. 5 min to adjust to the equipment and feel comfortable. When volunteers were inevitably interrupted from breathing into the MERDOC system for various reasons, e.g., sneezing, feeling of dried throat, getting on or alighting public transport, the inlet of the MERDOC system was closed manually (by applying clamp-on silicon tubing). In the data analysis, such occasions were detected by negative and noisy flowrate (due to flow direction change in the system). Such events were excluded from data analysis.

In this study, the breathing parameters (breathing rate and exhaled air flowrate), personal exposure, and RTD of BC were measured in 45 volunteers; however, only 40 (20 males and 20 females) study participants passed the initial data quality screening (some volunteers interrupted measurements too frequently, resulting in poor representation of investigated parameters). The demographics of the qualified study participants are summarized in Table 1.

**Table 1:** Demographics of male (n = 20) and female (n = 20) study participants

Unit		min	max	median	mean	SD	CI_95%_
Age (yr)	Male	19	27	20	21	2.4	1.1
	Female	18	27	20	20.8	2.6	1.2
Height (cm)	Male	149	175	165	166	5.7	2.7
	Female	142	165	158	157	4.8	2.3
Weight (kg)	Male	48	87	60.5	62.4	9.9	4.6
	Female	42	68	53.5	52.1	7.3	3.4

Here min, minimum; max, maximum; SD, standard deviation; CI_95%_, 95% confidence interval of the mean)

### Measurement quality control

To achieve high-quality measurement data, the procedures described in Madueño et al*.* [18] i.e., flow calibration, leak check, reference system check, particle loss estimation, instrument intercomparison (both in the laboratory and ambient setting, Additional file 1: Figs. S1–S3) were followed. The measured exposure and exhaled BC mass concentrations, as well as the flowrate of exhaled air were synchronized by lag cross-correlation. A thorough manual screening of each breath was done, flagging noisy data for exclusion in the subsequent data analysis. The accuracy of this method was previously validated by Madueño et al. [18].

### Data evaluation

The experimentally determined exhaled air flow rate, personal exposure, and exhaled air mass concentration of BC were used to calculate in situ deposition dose rate (DDR) following Eq. 1:

1 $${DDR }_{in situ}=\left({BC}_{in}-{BC}_{ex}\right)\times \underset{{t}_{0}}{\overset{{t}_{1}}{\int }}Qdt,$$

where *BC*_*in*_ and *BC*_*ex*_ are the BC mass concentrations (in μg m^−3^) of inhaled and exhaled air, respectively. Please note that here *BC*_*in*_ is assumed to be equal to the measured ambient BC mass concentration. The volume of inhaled air (in m^3^) was calculated from the flowrate measurements (Q, in L min^−1^) of exhaled air starting at (*t*_*0*_) and ending at (*t*_*1*_). In this study, the minute ventilation (MV) was calculated assuming inhaled air volume is equal to exhaled air volume. The total deposition fraction (DF) was defined as the percentage of the amount of BC mass concentration that was not expired after exhalation, generally stated as:

2 $$DF=(\left({BC}_{in}- {BC}_{ex}\right)/{BC}_{in})\times 100\%$$

The exposure to BC mass concentration, DDR, and DF was calculated in 1-min median values. Additionally, mean and standard deviation were calculated to compare this study’s results to previous work.

Data evaluation was performed using the R programming language and free software environment for statistical computing and graphics (R Core Team, 2015). The correlation analysis was calculated with Pearson’s product moment. The differences between groups were evaluated based on Wilcoxon rank sum test (also known as Mann–Whitney-U test) for independent variable comparisons, and Wilcoxon signed rank test for paired variable comparisons. The significances considered were those at the 0.01 level.

## Results and discussion

### General overview of BC exposure concentration in metro manila

**Fig. 3:**
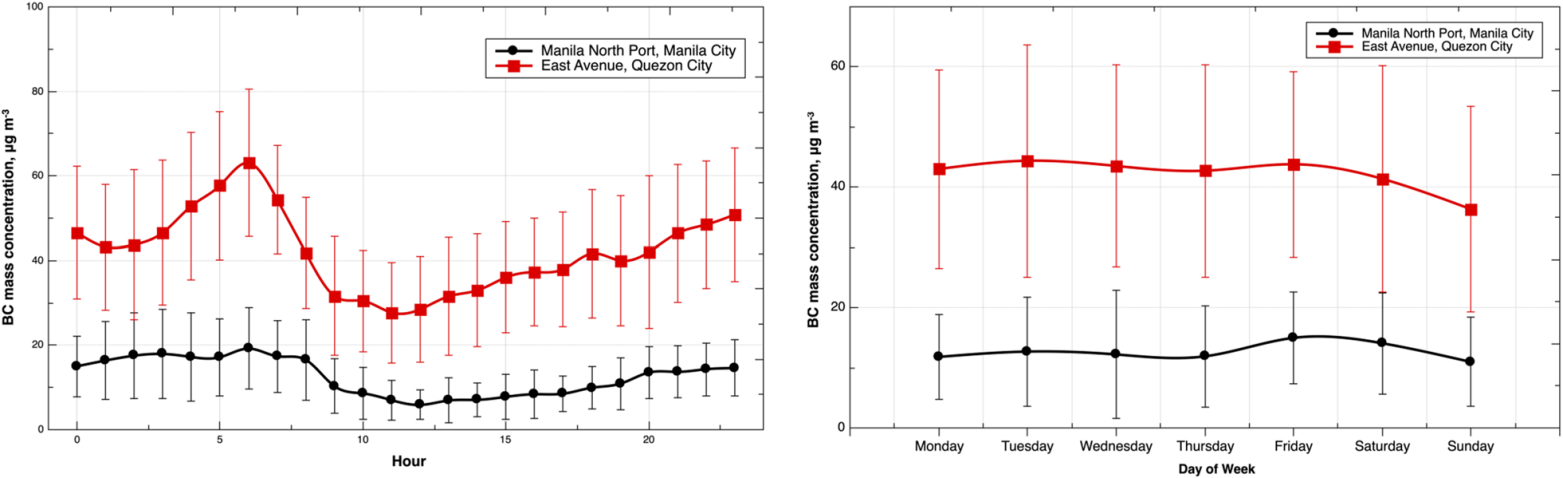
Diurnal and daily variation of BC mass concentration in Manila North Port, Manila City (red) and East Avenue, Quezon City (black). Error bars indicate standard deviation

During the TAME-BC campaign, the BC exposure concentration was assessed both at stationary sites and TMEs (i.e., public transportation and walking) in Manila and Quezon City. The temporal variation of BC measured at the stationary measurement sites (using multiple angle absorption photometer, MAAP; type 5012, Thermo Scientific Inc.) showed a pronounced diurnal pattern (Fig. 3), with the highest BC mass concentration during the morning rush hour and the lowest—during midday. The highest BC mass concentration (± standard deviation) was 19 ± 9.6 μg m^−3^ and 63.1 ± 17.4 μg m^−3^ in Manila North Port and East Avenue, respectively. There was no noticeable change in BC mass concentration between weekdays and weekends. A noticeably lower BC mass concentration was recorded in Manila North Port as a result of enhanced dilution from the nearby open water, versus the busy streetside with close proximity to the emission sources in Quezon City. Compared to other regions in the world, the BC seasonal average in Metro Manila is 25.7 ± 19.7 μg m^−3^ (dry season). This is 2 – 17 times higher than in urban and traffic sites in India (< 12 μg m^−3^) [34], China (< 5 μg m^−3^) [35], Europe (< 5 μg m^−3^) [36], and the USA (< 1.5 μg m^−3^) [37]. It must be noted that the BC mass concentration presented in this study corresponds to 5 months of BC mass concentration measurements in the Philippines. The annual average remains unknown.

**Fig. 4:**
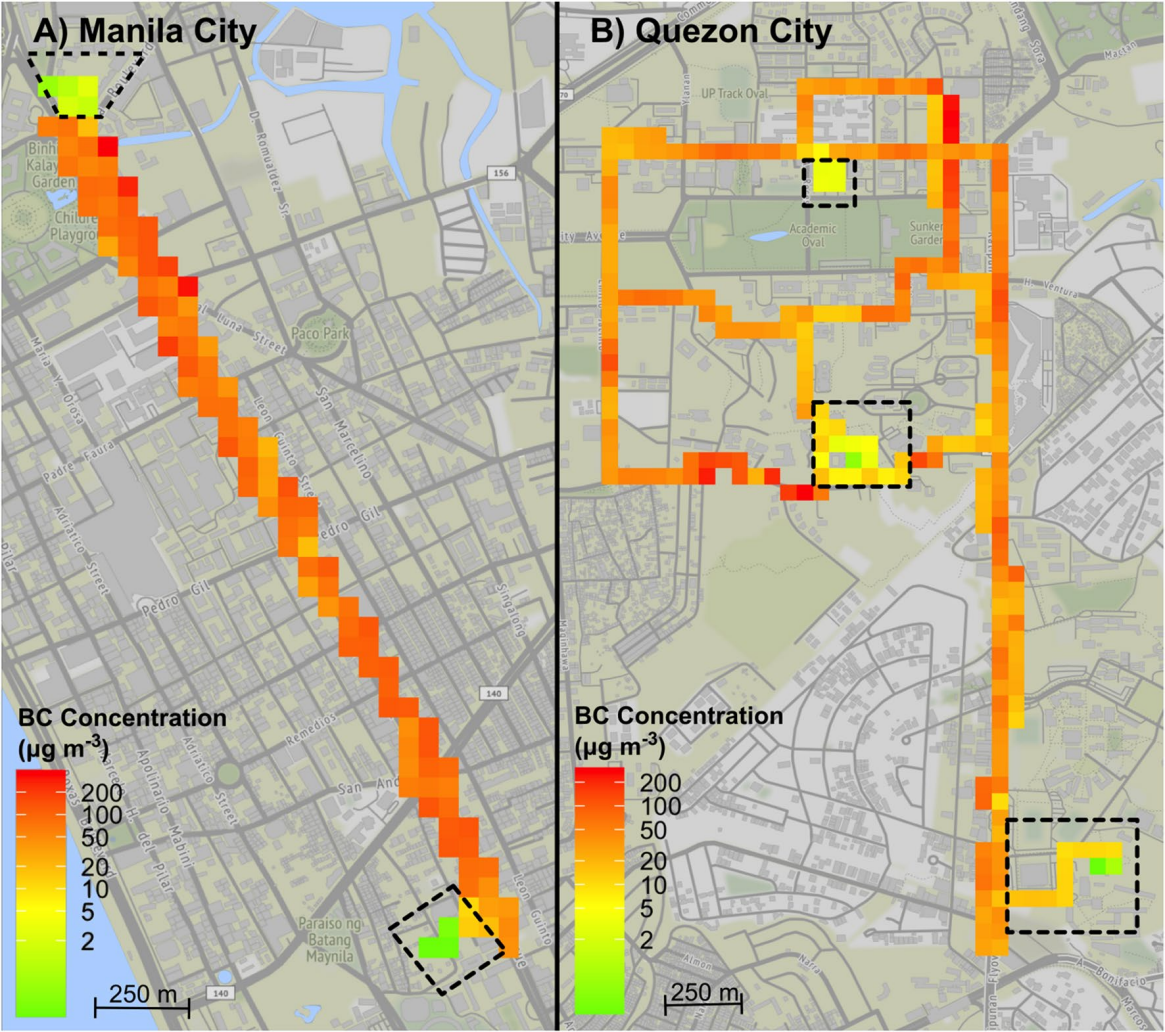
Spatial distribution of BC mass concentration in TMEs along the routes in **A** Manila and **B** Quezon City. Dashed lines correspond to an inner gated area with restricted traffic. The midpoint of the color scale was set to 15 μg m^−3^ (yellow) to visually represent the areas where the BC mass concentration alone exceeded (orange to red) the World Health Organization suggested PM_2.5_ daily limit values. Each tile is a 55 m square grid resolution

Mobile measurements of BC exposure concentration in TMEs showed an average value of 50.0 ± 62.6 μg m^−3^ (average for Manila City: 57.5 ± 68.7 μg m^−3^; and average for Quezon City: 36.7 ± 47.1 μg m^−3^). The observed exposure concentration in TMEs is up to twofold higher compared to values reported from stationary measurement sites. It may suggest that substituting mobile measurements with stationary sites in exposure assessment may not represent true exposure concentrations, especially during transportation. The spatial distribution of BC mass concentration in TMEs between cities is shown in Fig. 4.

Although the exposure experiment was focused on riding public transport and walking, there is an apparent difference in the BC exposure in TMEs between the cities. The discrepancy may be explained by the effect of street topography and micro-meteorology. The commuting route in Manila city was located on a major thoroughfare. Both sides of the street are occupied by tall buildings resulting in a deep street canyon geometry [38] (street aspect ratio (height/width) of approx. 2). Such a setting influences poor airflow across the street, thus, increasing the accumulation of pollutants. While in Quezon City, the commuting mostly took place on an open-spaced road [38] (street aspect ratio of less than 0.5).

### Effect of activity on respiratory tract deposition

The descriptive statistics of MV, breathing rate (BR), tidal volume (TV), BC exposure concentration, DDR, and DF in different modes of transportation are summarized in Table 2 and Additional file 1: Fig. S5. The median BR values while riding public transport and slow walking, were 11.8 and 13 breaths min^−1^, respectively. The observed TV both resulted in 0.6 L breath^−1^, while the MV was 7.5 L min^−1^ and 8.1 L min^−1^ during public transport and walking, respectively. The effect of commuting mode on all breathing parameters was small and showed no statistically significant difference (*p* value > 0.01). A possible reason for the similarity in the breathing patterns between sitting inside public transport and light exercise (walking) is due to the configuration of passenger seats inside the *Jeepney*. The seats are not as comfortable as in a regular vehicle, and the orientation of passengers is seated sideways (Fig. 1a). This means that for every acceleration or deceleration of the PUV, the passengers must compensate for their sitting position, which may have caused slight physical activity similar to slow walking.

**Table 2:** Summary of descriptive statistics for different exposure scenarios in public transport (PT) and walking (W)

Variable	MV		BR		TV		BC		DDR		DF	
Unit	L min^−1^		# min^−1^		L		μg m^−3^		μg hr^−1^		%	
	PT	W	PT	W	PT	W	PT	W	PT	W	PT	W
min	1.6	1.3	7	7	0.14	0.16	23.3	4.2	0.85	0.20	20.3	16.6
max	16.9	17.3	28	22	1.42	1.63	128	119	37.3	21.2	79.1	82.6
median	7.5	8.1	11.8	13	0.63	0.61	71.5	30	11.6	4.4	39.3	43.9
mean	7.6	7.8	12.9	12.9	0.63	0.64	72.4	34.2	13.2	6.3	41.1	45
SD	4.0	3.9	4.7	3.9	0.34	0.37	30	25.5	9.3	5.3	10.7	11.7
CI	1.3	1.3	1.5	1.2	0.11	0.12	9.6	8.15	2.3	1.7	3.43	3.8
*p *value	> 0.01		> 0.01		> 0.01		< 0.01		< 0.01		< 0.01	
effsize r	0.1 (small)		0.07 (small)		0.2 (small)		1.0 (large)		0.9 (large)		0.5 (medium)	

Here, SD, standard deviation; CI, 95% confidence interval of the mean; *p* value calculated from the Wilcoxon signed rank test; effsize, r rank biserial)

The BC mass concentration was more than twofold higher while commuting in PUV compared to walking (72 vs. 30 μg m^−3^, respectively). The DF of BC between different commuting modes was noticeably different (39% for a ride in public transport versus 44% for a commute by walking). The respiratory tract DDR of BC while riding PUV and walking was 12 μg hr^−1^ and 4 μg hr^−1^, respectively. The choice of commuting mode showed a statistically significant difference (*p* value < 0.01) between the BC exposure concentration, DF, and respiratory tract DDR. Suggesting that the respiratory tract DDR is primarily determined by respectively higher exposure concentrations of BC inside PUV compared to walking.

The measured breathing parameters, i.e., BR, TV, MV, in this study are comparable with previous studies (± 10%) where respiratory tract deposition of aerosol BC particles during spontaneous breathing on healthy subjects was measured (Table 3). However, if comparing the measured MV values to those reported in EPA Exposure Handbook [39], we can see that it is significantly different. Therefore, using standard reported values might result in errors when estimating respiratory tract deposition of Filipinos (underestimation when using MV for sitting; overestimation when using MV for exercise). Contrary to EPA Exposure Handbook, standard values reported by the International Commission on Radiological Protection (ICRP) [40] shows better agreement between reported and measured MV values (except for MV during exercise). The differences between measured and reported MV values may occur due to several reasons, e.g., considered range of metabolic activities, anthropometric and anatomical disparities of respiration related to ethnicity [41, 42]. Study design and measurement set-up to determine respiratory parameters could also influence the results (for further discussion, please refer to section Study Considerations). We thus recommend using MV values from this study or those reported by ICRP (while at rest) when calculating respiratory tract deposition of airborne pollutants in the Filipino population. For the studies, where breathing rate data relevant to the population considered is not available, authors shall conduct a sensitivity study evaluating the effects of the chosen breathing parameters onto respiratory tract deposition.

**Table 3:** Reported breathing parameters of healthy subjects from similar experimental studies

Study	Activity	Age	Subjects	BR	TV	MV
		(yrs)	(n)	(# min^−1^)	(L breath^−1^)	(L min^−1^)
U.S. EPA[39]	Sitting	21–31	1724	–	–	4.2
	Exercise^a^	21–31	1724	–	–	12
ICRP [40]	Sitting	20–50	–	13 ± 1.4	0.6 ± 0.2	7.8 ± 1.8
	Exercise	20–50	–	21 ± 0.7	1.2 ± 0.2	24 ± 2.1
Tobin et al*.* [43]	Resting	23–34	47	17 ± 2.7	0.4 ± 0.1	6.2 ± 1.3
Daigle et al*.*[21]	Sitting	18–52	12	16 ± 2.8	0.6 ± 0.1	9.0 ± 1.3
	Exercise^b^	18–33	7	29 ± 5.4	1.3 ± 0.4	38 ± 10
Löndahl et al*.*[17]	Sitting	22–34	28	12 ± 2.0	0.7 ± 0.2	7.8 ± 1.5
Löndahl et al. [23]	Sitting	20–40	29	12 ± 2.3	0.7 ± 0.2	7.8 ± 1.5
	Exercise^c^	20–40	29	17 ± 3.7	2.1 ± 0.5	34 ± 8.0
Löndahl et al*.* [22]	Sitting	21–31	10	12 ± 2.1	0.7 ± 0.2	8.1 ± 2.8
Löndahl et al*.* [27]	Sitting	21–38	9	11 ± 1.6	0.7 ± 0.2	7.6 ± 1.2
Rissler et al*.* [44]	Sitting	23–45	9	10 ± 3.6	0.9 ± 0.3	9.0 ± 4.4
Rissler et al*.* [26]	Sitting	20–29	19	10 ± 3.6	0.7 ± 0.2	7.3 ± 2.2
Guo et al*.* [45]	Sitting	34	1	20	0.4	8.0
This study	Sitting	18–27	40	13 ± 6	0.6 ± 0.4	7.7 ± 4.6
	Exercise^d^	18–27	40	14 ± 8	0.6 ± 0.4	7.8 ± 4.6

Values from widely used exposure EPA Exposure Handbook and ICRP Reference Values are added for comparison

Mean ± SD; a = light intensity 1.5 < METS < 3.0; b = 15 min. of moderate exercise in ergometer; c = light exercise at 65–75% of estimated maximal heart rate; d = slow walking, average speed of 3.5 km h^−1^

The measured DDR of BC in the respiratory tract of multiple subjects showed high variability, which is a result of changing exposure concentrations and subject-specific breathing parameters (Additional file 1: Fig. S7). The comparison of experimentally-measured RTD of hydrophobic particles segregated by activity (e.g., sitting, exercise) is presented in Additional file 1: Table S2, and the comparison between different assessment methods is summarized in Additional file 1: Table S3. Overall, the TV, BR, and exposure concentration of BC are some of the most important variables to estimate RTD. The breathing parameters are especially crucial when simulating RTD using dosimetry model, i.e., freely available Multiple-Path Particle Dosimetry model (MPPD [16]; (http://www.ara.com/products/mppd.htm)).

### Effect of gender on respiratory tract deposition

The gender separated breathing parameters, BC exposure concentration, respiratory tract DDR, and DF are summarized in Table 4 and Additional file 1: Fig. S6. The MV, BR, and TV showed no statistically significant difference (*p* value > 0.01) and were 7.2 versus 6.9 L min^−1^, 11.8 versus 12.5 breath min^−1^, and 0.7 versus 0.6 L breath^−1^ for males and females, with a small to medium effect size (rrb = 0.1, 0.2, and 0.3), respectively.

**Table 4:** Summary of descriptive statistics segregated between females (F) and males (M).

Variable	MV		BR		TV		BC		DDR		DF	
Unit	L min^−1^		# min^−1^		L		μg m^−3^		μg hr^−1^		%	
	F	M	F	M	F	M	F	M	F	M	F	M
min	1.6	2.2	7.0	7.5	0.14	0.23	22.6	21.1	1.0	1.4	26.4	20.3
max	12.6	17.1	24.5	22	1.3	1.4	124	110	26.7	23.5	59.8	81.7
median	6.9	7.2	12.5	11.8	0.55	0.73	69.8	57.0	7.7	8.2	39.7	41.4
mean	7.2	8.1	13.4	12.5	0.55	0.70	65.6	58.4	10.8	9.8	41.4	42.1
SD	3.5	4.0	4.2	4.4	0.29	0.37	29.5	25.9	7.6	6.2	8.3	13.5
CI	1.7	1.9	1.9	2.1	0.14	0.17	3.9	12.1	3.5	2.9	3.9	6.3
*p-value*	> 0.01		> 0.01		> 0.01		> 0.01		> 0.01		> 0.01	
effsize r	0.1 (small)		0.2 (small)		0.3 (medium)		0.2 (small)		0.1 (small)		0.01 (small)	

SD, standard deviation; CI, 95% confidence interval of the mean; *p* value calculated from the Wilcoxon rank sum test; effsize, r rank biserial

The BC exposure of females was at 69.8 μg m^−3^, while males were at 57 μg m^−3^. Although the comparison between the exposure scenario showed no statistically significant difference between genders (*p* value > 0.01), the effect size is small (rrb ≤ 0.2). This means that in spite of the randomization of the exposure scenario, the erratic and high irregularities in traffic may have affected sporadic BC exposure of study participants. Interestingly, the corresponding respiratory tract DDR and DF of BC showed no statistically significant difference for males and females (*p* value > 0.01, 8.2 versus 8.3 μg hr^−1^, and 42% versus 40%, respectively). Nevertheless, the results show that despite the differences in the physiology of males and females (e.g., males have slightly higher TV but lower BR contrariwise to females), the practical effect is small (rrb ≤ 0.2). Each factor may balance each out, resulting in similar DDR and DF between genders. Similar results were also reported by Löndahl et al. [23]. Moreover, the experimental and computational studies of Kim and Jacques [46], and Sturm [47] also reported essentially comparable total DF values between males and females for inhaled particles with a diameter greater than 80 nm.

### Study considerations

There are several limitations to the MERDOC measurement system based on its design aspects [18]. Firstly, the measurement can only determine the total mass deposition of BC particles in the entire respiratory tract, thus the information on regional lung deposition is unknown. Because of this, much desired dosimetry model evaluation based on experimental respiratory tract deposition measurements is not possible at this moment. To enable such model validation, ambient and exhaled BC PNSD must be known. This requires mobile aerosol instrumentation, capable to determine either PNSD and the mixing state of aerosol particles (BC PNSD can then be calculated) or BC PNSD itself. Although such instrumentation exists for stationary measurements, its portability to this moment is seriously limited. In general, one may try to estimate BC PNSD based on BC mass concentration measurements, assuming geometric mean diameter and standard deviation of BC particles, constraining reconstructed BC PNSD by the total measured mass of BC particles, however this requires a number of critical assumptions and is beyond the scope of this study. Moreover, in such way determined regional DF would be more of qualitative nature rather than means to validate dosimetry model. Stationary measurements could provide some advantage when estimating BC PNSD, however, neither in this study nor in previous works BC PNSD were determined in variety of environments covering multiple commuting scenarios. Secondly, for the exhaled BC concentration measurements, subjects had to inhale exclusively through nose, followed by exhalation through mouth into mouthpiece. Such measurement set-up might result in deviation from normal physiological breathing pattern, which in turn might influence determined minute ventilation, as well as respiratory tract deposition. With respect to respiratory deposition, the dominating mechanism for lung deposition of nanoparticles with diameter less than 500 nm is diffusion, which primarily takes place in the acinus (alveolar region; due to extended residence time). As hydrophobic BC particles have a mean geometric diameter of approx. 80–200 nm [30], their growth shall be limited during the inhalation and exhalation cycle in the respiratory tract. This means that deposition in the upper airways or the increased filtration efficiency through nasal breathing shall not significantly affect the BC deposition dose levels among mouth or nose breathers [48]. It can also be demonstrated that mouth breathing and combined breathing show similar flow profiles, hence, the inhalation route does not affect the distribution of particles in the lower airways [49]. It must be noted, however, that given the measurement set-up, which was optimized for mobile measurements and not standard laboratory lung function test, the measured tidal volume could deviate from real values. In the future, a comparison between MERDOC and more standardized systems (e.g., RESPI [17]) could help to further validate mobile respiratory tract deposition measurement set-up for field experiments.

Another limitation in this study is with regard to the experimental procedure. Although this study showcased one of the largest real-world in situ measurements of respiratory tract deposition in terms of the number of study participants, it was only limited to young healthy adults. Investigations of respiratory tract deposition in children, the elderly, and people with lung disorders could provide comprehensive information. The sampled breath of study participants, though instructed to be as natural as possible, was to a certain degree disturbed due to the use of a mouthpiece. Some experiments are using a similar technique, however, a thorough consideration of the experimental method must be noted when comparing breathing patterns and respiratory deposition dose with other studies.

## Summary and conclusion

Establishing an exposure-dose relationship is critical to better understand the health response of inhaling particulate matter. Among several different methods to determine respiratory tract deposition dose, in situ measurements, albeit complicated, are arguably the best one, as it accounts for the complexity of the human respiratory system, ambient conditions, and particle physico-chemical properties. In this study, the respiratory tract deposition of BC particles under real-world conditions in the developing megacity of Metro Manila was investigated. The experiment was successfully conducted with 40 young, healthy adults during their commute by riding public transport and walking.

The observed roadside BC exposure concentration in this study is up to 17 times higher than in other studies [36, 37], and the deposition dose of BC is up to twice higher than in a comparable study [22]. The study results showed that the mode of transportation, i.e., sitting inside public transport versus slow walking, had no significant influence on the BR, TV, and MV. The breathing parameters between genders also showed no statistically significant difference. Conversely, the respiratory tract deposition dose rate of BC is significantly higher in public transport than walking (Wilcoxon signed rank test *p*-value < 0.01). This is due to correspondingly higher BC exposure concentration inside public transport (72 μg m^−3^ versus 30 μg m^−3^, respectively). Thus, it would be advisable to choose walking as a mode of transportation for shorter trips, especially if this includes routes further away from the roadside. Regular users of public transport (*Jeepney*) should consider wearing high-quality facemasks equipped with high-efficiency particulate filters to reduce exposure. The comparison between measured true DDR of BC in different modes of transportation and alternative methods to estimate RTD highlights the importance of subject-specific breathing parameters and PNSD in the dosimetry calculations. Furthermore, miniaturization of current stationary aerosol instrumentation for PNSD and BC PNCD measurements would enable mobile in situ determination of both ambient and exhaled PNSD, which could be used for comprehensive dosimetry model evaluation.Additional file 1.

This study is the first attempt toward assessing the carbonaceous pollution-related health effects through the systematic in situ determination of real-world respiratory tract deposition dose of BC in developing regions. The provided information on respiratory tract deposition and breathing parameters can be used to advance epidemiological assessment of health risks of carbonaceous pollution.

## Supplementary Information

**Additional file 1**. Experiment quality assurance and supplementary results. **Table S1:** List of related in situ respiratory tract deposition dose studies. **Table S2:** Summary of related in situ respiratory tract deposition studies using hydrophobic particles. **Table S3:** Mean DDR estimated using different assessment methods. **Figure S1:** Instrument laboratory intercomparison with reference system. **Figure S2:** Micro-aethalometer intercomparison in Leipzig, Germany. **Figure S3:** Micro-aethalometer intercomparison in Metro Manila, Philippines, using ambient street-site aerosol. **Figure S4:** Flow rate through dry and wet (after exposing to breath air) particulate filter. **Figure S5:** Descriptive statistics of measured parameters in TMEs between public transport and walking. **Figure S6:** Descriptive statistics of measured parameters separated between males and females. **Figure S7:** Deposition dose rate as a function of measured BC exposure concentrations.

## Data Availability

Processed and raw data are available upon request from the corresponding author.

## Abbreviations

BC: Equivalent black carbon
DF: Deposition fraction
DDR: Deposition dose rate
RTD: Respiratory tract deposition
PM: Particulate matter
TME: Transport microenvironment
LMIR: Low-to-middle income regions
TAME-BC: Transdisciplinary approach to mitigate emissions of black carbon
