# Supplementary material for: A novel in-situ method to determine the respiratory tract deposition of carbonaceous particles reveals dangers of public commuting in highly polluted megacity
Source: Part Fibre Toxicol. 2022 Sep 15;19:61. doi: 10.1186/s12989-022-00501-x (PMC9476571; doi:10.1186/s12989-022-00501-x)
Supplement: Supplementary file 1 — Additional file 1. Experiment quality assurance and supplementary results. Table S1: List of related in situ respiratory tract deposition dose studies. Table S2: Summary of related in situ respiratory tract deposition studies using hydrophobic particles. Table S3: Mean DDR estimated using different assessment methods. Figure S1: Instrument laboratory intercomparison with reference system. Figure S2: Micro-aethalometer intercomparison in Leipzig, Germany. Figure S3: Micro-aethalometer intercomparison in Metro Manila, Philippines, using ambient street-site aerosol. Figure S4: Flow rate through dry and wet (after exposing to breath air) particulate filter. Figure S5: Descriptive statistics of measured parameters in TMEs between public transport and walking. Figure S6: Descriptive statistics of measured parameters separated between males and females. Figure S7: Deposition dose rate as a function of measured BC exposure concentrations. [file 12989_2022_501_MOESM1_ESM.docx]

**Supplementary Material**

**A Novel in-situ Method to Determine the Respiratory Tract Deposition of Carbonaceous Particles Reveals Dangers of Public Commuting in Highly Polluted Megacity**

***Leizel Madueño^1^, Simonas Kecorius^1,5*^, Jakob Löndahl^2^, Jürgen Schnell-Kreis^3^,***

***Alfred Wiedensohler^1,4^, Mira Pöhlker^1^***

^1^ Experimental Aerosol and Cloud Mircophysics, Leibniz-Institute for Tropospheric Research, Leipzig, Germany
^2^ Ergonomics and Aerosol Technology, Lund University, Lund, Sweden

^3^ Comprehensive Molecular Analytics, Helmholtz Zentrum München—German Research Center for Environmental Health, München, Germany

^4^ World Calibration Center for Aerosol Physics, Leipzig, Germany

^5^ currently at Institute of Epidemiology, Helmholtz Zentrum München—German Research Center for Environmental Health, Neuherberg, Germany

Corresponding Author:

Simonas Kecorius (simonas.kecorius@helmholtz-muenchen.de)

Current Affiliation: *Institute of Epidemiology, Helmholtz Zentrum München, Ingolstädter*

*Landstr. 1, 85764, Neuherberg, Germany*

Contents of this Supplementary Material

Table S1. List of related *in situ* respiratory tract deposition dose studies.

Table S2. Summary of related in situ respiratory tract deposition studies using hydrophobic particles.

Table S3. Mean DDR estimated using different assessment methods.

Figure S1. Instrument laboratory intercomparison with reference system.

Figure S2. Micro-aethalometer intercomparison in Leipzig, Germany.

Figure S3. Micro-aethalometer intercomparison in Metro Manila, Philippines, using ambient street-site aerosol.

Figure S4. Flow rate through dry and wet (after exposing to breath air) particulate filter.

Figure S5. Descriptive statistics of measured parameters in TMEs between public transport and walking.

Figure S6. Descriptive statistics of measured parameters separated between males and females.

Figure S7. Deposition dose rate as a function of measured BC exposure concentrations.

Table S1. Review of related literature. There are few *in situ* studies on respiratory tract deposition (RTD) conducted in developed regions, but almost none have investigated roadside ambient air and none of the studies have focused on black carbon particles. The discussion in the main manuscript was focused only on the related studies where total mass RTD of hydrophobic particles was investigated on young and healthy adults.

|  | Location | Subjects | Age, Health Status | Aerosol | Breathing | Particle Detection |
| --- | --- | --- | --- | --- | --- | --- |
| Daigle et al., [1] | USA | 11M/8F | 18-52, Healthy | Spark discharge | Mouth, Spontaneous | SMPS, CPC |
| Chalupa *et al.*, [2] | USA | 8M/8F | 18-55, Asthma | Spark discharge | Mouth, Spontaneous | SMPS |
| Morawska *et al.*, [3] | Australia | 8M/6F | 20-30, Non-smoker | Combustion | Nose, Spontaneous | SMPS |
| Londahl *et al.*, [4] | Sweden | 3M | 26-31, Healthy | NaCl; DEHS | Mouth Spontaneous | SMPS |
| Londahl *et al.*, [5] | Sweden | 19M/9F | 23-34, Healthy | NaCl; DEHS | Mouth Spontaneous | SMPS |
| Londahl *et al.*, [6] | Sweden | 4M/6F | 21-31, Healthy | Biomass combustion; DEHS | Mouth Spontaneous | SMPS |
| Londahl *et al.*, [7] | Sweden | 5M/4F | 21-38, Healthy | Street | Mouth, Spontaneous | SMPS |
| Jakobsson *et al.*, [8] | Sweden | 5M/2F | 20-34, Healthy | PSL | Single breath | CPC |
| Rissler *et al.*, [9] | Sweden | 25M/35F | 7-70, Healthy | Carnuba wax particles, glass particles | Mouth, Spontaneous | SMPS, APS |
| Lin *et al.*, [10] | Taiwan | 10M/2F | 22-37, Healthy | DEHS | Nose; Mouth | CPC |
| Madueno *et al.*, [11] | Bolivia | 1M | 35, Healthy | Roadside | Nose; Mouth, Spontaneous | Light absorption |
| Guo *et al.*, [12] | Australia | 63M/65F | 8-11, Healthy | Roadside;  Urban Background | Mouth, Spontaneous | SMPS |
| This study | Philippines | 20M/20F | 18-27, Healthy | Roadside | Nose; Mouth, Spontaneous | Light absorption |

NaCl: Sodium chloride

DEHS: Di(2-ethylhexyl) sebacate

PSL: Polystyrene Latex

Table S2. Summary of related in situ respiratory tract deposition studies using hydrophobic particles. DF presented in Mean ± SD; C_in_ is the exposure mass concentration used in the respective experiments. DD is the deposition dose.

| Study | Aerosol Type | Breathing Pattern | Age | Subjects* | Activity | Mass DF*_in situ_**, % | C_in_, μg m^-3^ | Norm. DD^§^, μg | |
| --- | --- | --- | --- | --- | --- | --- | --- | --- | --- |
| Daigle *et al.* [1] | Spark discharge | Nose clipped, mouth breathing;  Spontaneous | 18-52 | 6M/6F  12 | sitting | 60 ± 13 / 59 ± 14 | 10 | 32.1 / 31.9 | |
|  |  |  |  |  | sitting | 58 ± 13 | 10 | 31.3 | |
|  |  |  | 18-33 | 7  7 | sitting | 60 ± 4 | 10; 25 | 32.4 | |
|  |  |  |  |  | exercise | 76 ± 6 | 10; 25 | 174.0 | |
| Löndahl *et al.*[6] | Efficient biomass combustion | Nose clipped, mouth breathing;  Spontaneous | 21-31 | 10 | sitting | 24 ± 7 | 130; 250 | 11.8 | |
| Löndahl *et al.*[7] | Ambient Traffic Exhaust | Nose clipped, mouth breathing; Spontaneous | 21-38 | 5M/4F | sitting | 28 ± 3 | 40 (PM_10_) | 12.9 | |
|  |  |  |  |  |  |  |  |  | |
| Rissler *et al.*[13] | Diesel Exhaust Particles | Nose clipped, mouth breathing;  Spontaneous | 23-45 | 10 | sitting | 27 ± 7 | 300; 59, diluted 100-250 times | 14.6 | |
|  |  |  |  |  |  |  |  |  | |
| Rissler *et al.*[9] | Carnauba wax /glass particles | Nose clipped, mouth breathing;  Spontaneous | 20-70 | 60 | sitting | 34 ± 8 | - | 15.6 | |
| Muala *et al.*[14] | Wood stove smoke | Nose clipped, mouth breathing;  Spontaneous | 21-27 | 12 | sitting | 22 ± 6 | 81 ± 35; 132 ± 26 | 10.9 | |
|  |  |  |  |  |  |  |  |  | |
| This study | Ambient Traffic Exhaust | Nose inhalation, mouth exhalation; Spontaneous | 18-27 | 20M/20F | mobile** | 44 ± 18 / 42 ± 14 | 2-375; actual ambient | 20.2 / 19.3 | |
|  |  |  | 18-27 | 40 | mobile** | 43 ± 16 | 2-375; actual ambient | 19.8 | |
| *Number of subjects separated between Male/Female, or total number  **Combined data for commute while sitting and light walking due to statistically similar values.  ^§^Normalized deposition dose calculated for a mass concentration of 100 μg m^-3^, and an exposure time of 1 h. | | | | | | | | |  |

Comparing our observed results to other studies needs a careful interpretation due to different circumstances in each experiment design, e.g., study participants’ age range, characteristics of sampled breath, etc. For *in situ*-determined RTD, only limited studies exist, thus, the comparison is even more challenging. The results of aerosol *in situ* studies focused on investigating the deposition fraction (DF*_in situ_*) of different hydrophobic particles in healthy adults are presented in Table S2. It is important to note that different experimental techniques were used in each study. Most of the previous studies were performed in controlled laboratory exposure scenario. In this study, the DF was found to be 43 ± 16%, which is 25% less than the DF observed by Daigle et al. [1], but almost twice as high as values reported in some other studies. The study by Löndahl et al. [7] closely resembles the experiment design of this study and has reported similar measured MV values (Table 3). However, DF found by Löndahl et al. [7] was 28%, which is 1.5 times lower than observed in this study. One possible reason for this might be differences in the physical properties of exposure particles (e.g., PNSD). Another, more complex reason for observed differences in DF may arise from the different physiology of the study participants, subjective health status, and possible effects of the limited number of study participants.


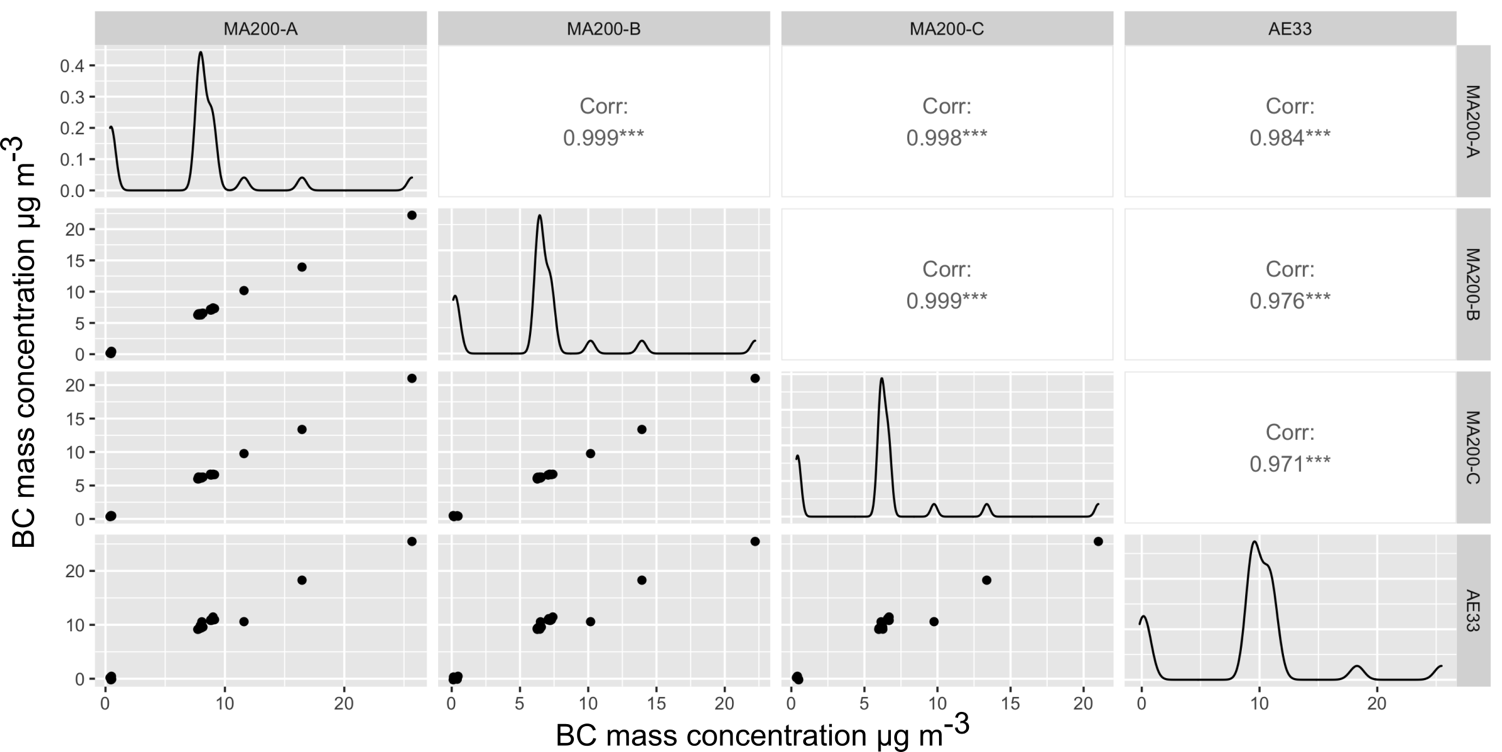


Figure S1. Instrument laboratory intercomparison with reference system. The MA200-A was used to measure BC mass concentration of ambient air, MA200-B and MA200-C for exhaled BC in MERDOC 1 and MERDOC 2, respectively. AE33 (Magee Scientific Aethalometer ®, Model AE33) was used as a reference system. The correlations displayed are Pearson’s correlation coefficient.


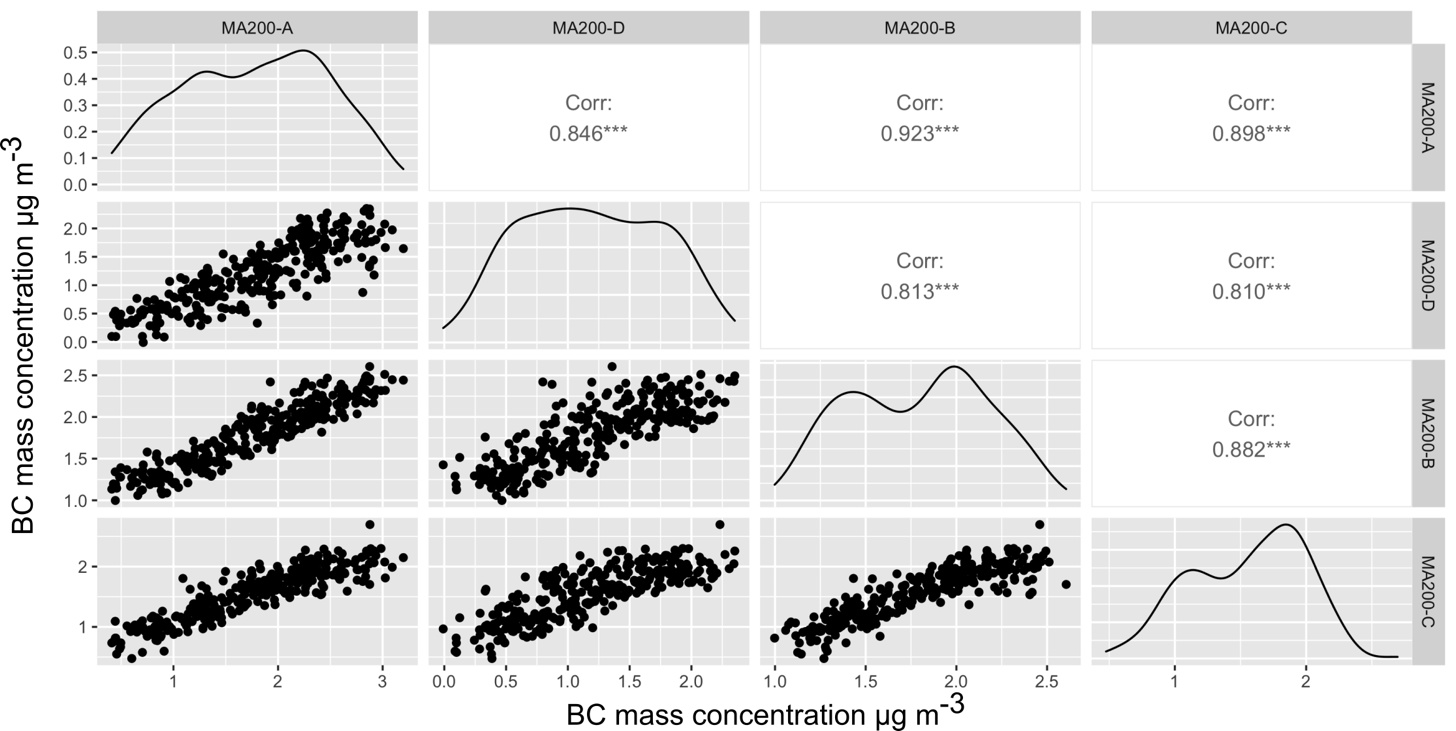


Figure S2. Micro-aethalometer intercomparison in Leipzig, Germany. The MA200-A was used to measure equivalent black carbon (BC) mass concentration of ambient air, MA200-B and MA200-C for exhaled BC in MERDOC 1 and MERDOC 2, respectively. MA200-D was prepared as a reserve instrument (not used in this study). The correlations displayed are Pearson’s correlation coefficient.


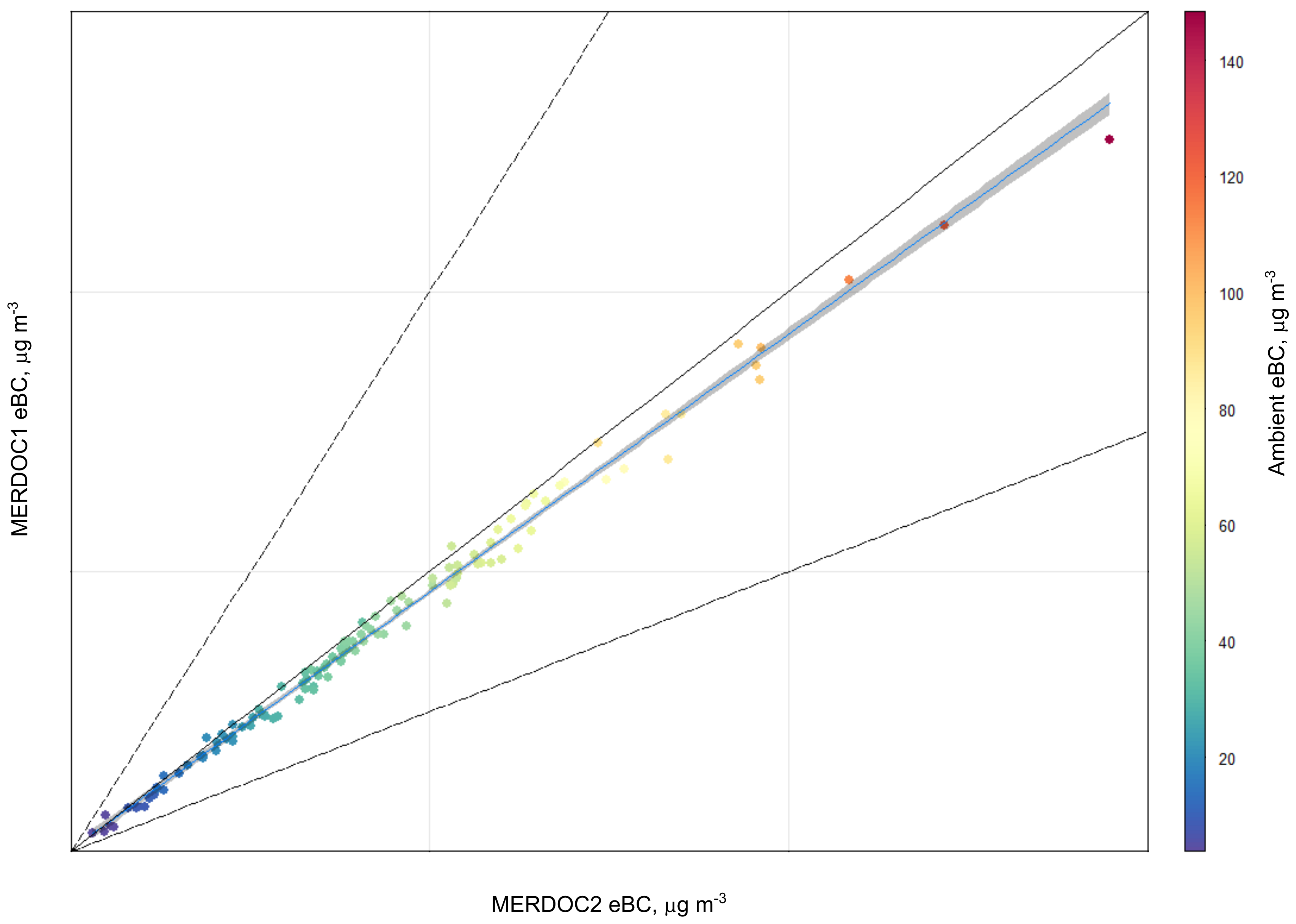


Figure S3. Micro-aethalometer intercomparison in Metro Manila, Philippines, using ambient street-site aerosol. MERDOC1 = 0.92 x MERDOC2 + 0.34 (R^2^ = 0.99); Ambient = 1 x MERDOC2 – 0.18 (R^2^ = 0.99); Ambient = 1.1 x MERDOC1 – 0.34 (R^2^=0.99)


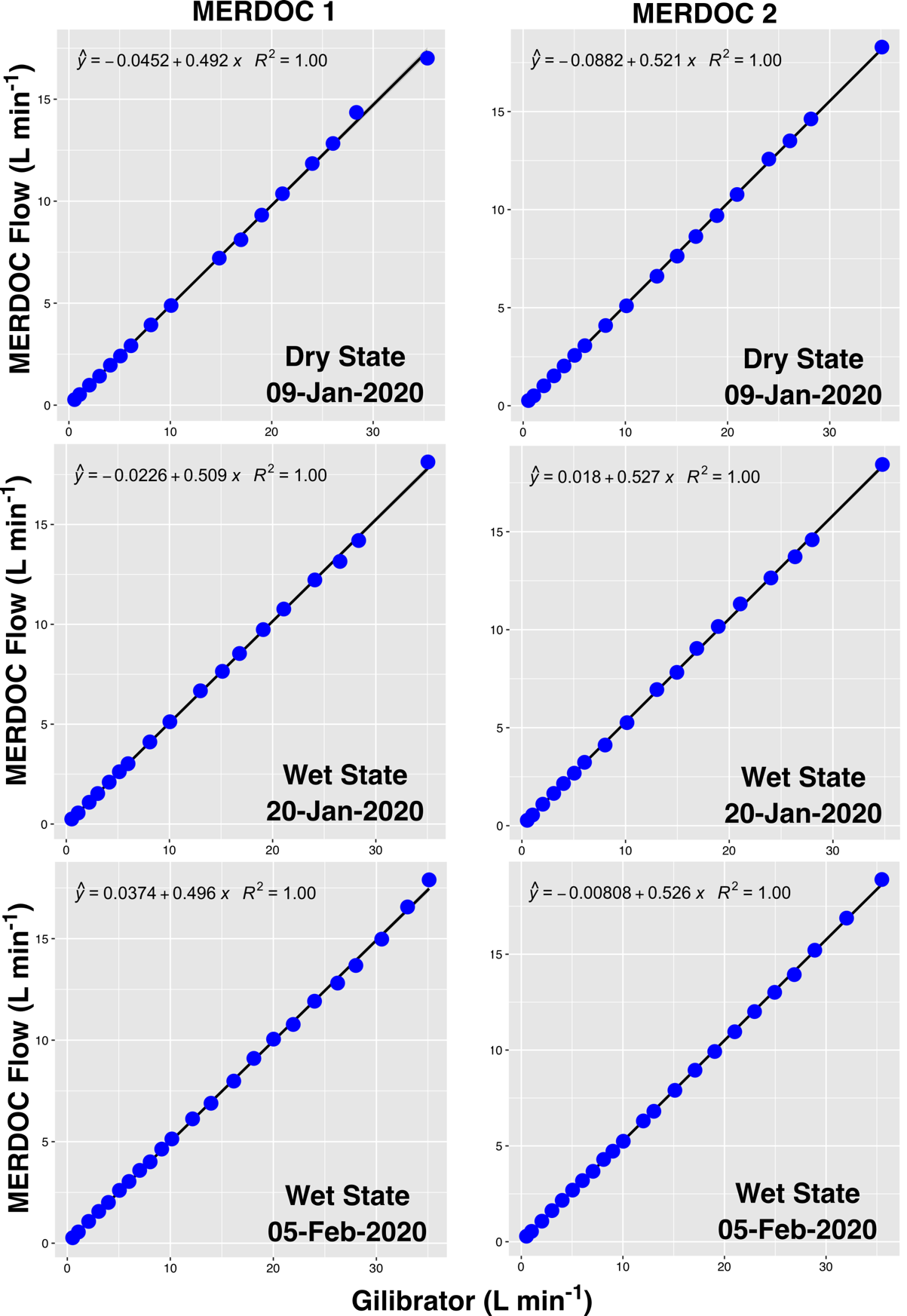


Figure S4. Flow rate through dry and wet (after exposing to breath air) particulate filter. No change in flow rate was observed with respect to HEPA filter wetting.


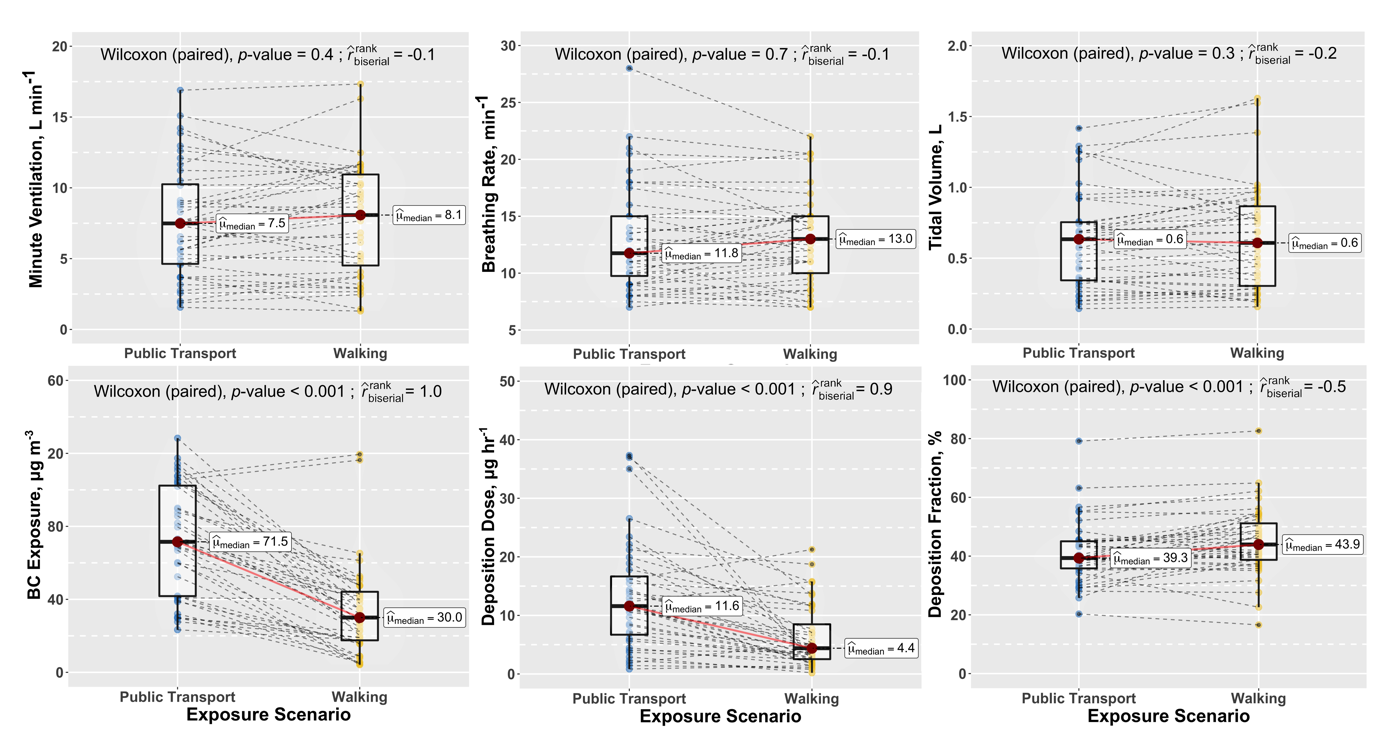


Figure S5**.** Descriptive statistics of measured parameters in TMEs between public transport and walking**.** The data points represent the median value of study participants (n = 40) for each parameter. The *p*-values were calculated from the Wilcoxon signed rank test at 0.01 significance level. The effect size (r rank biserial, rrb) was interpreted to be small when the absolute value is ≤ 0.2. The rrb < 0 indicates that the measured parameters tend to be higher when the study participants chose to commute by walking than using public transport. There is a statistically significant difference in the DF when a commuter chooses between public transport or walking (p-value < 0.01). There is a practical medium effect that the DF tends to be higher during commute by walking (rrb = -0.5) than the use of public transport.

**
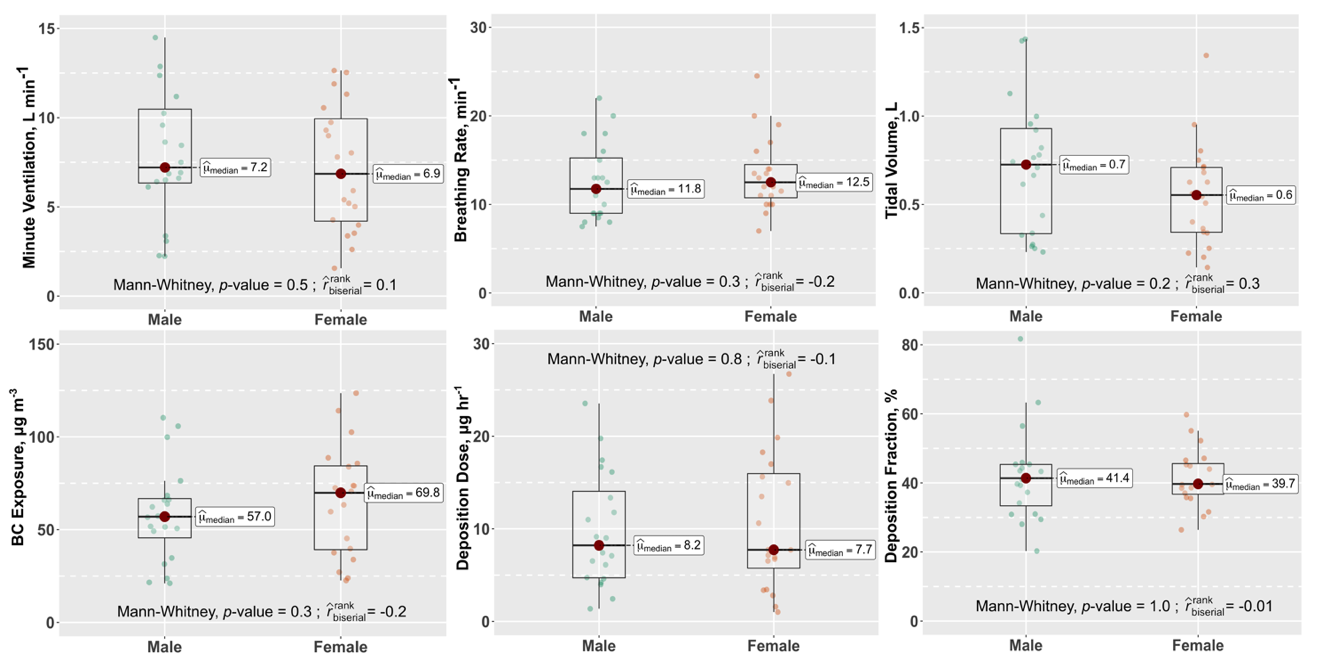
**

Figure S6. Descriptive statistics of measured parameters separated between males and females. The *p*-values indicated are results from the Wilcoxon rank sum test (also known as Mann-Whitney-U test) at 0.01 significance level. The data points represent the median value of study participants (n = 40) for each parameter.

***Importance of Breathing Parameters to RTD Assessment***

To illustrate the importance of subject-specific breathing rates, BC particle number size distribution (PNSD), and exposure concentrations in assessing RTD, we calculated DDR using previously published methods and compared the results to experimentally measured values in this study (Table S3). Firstly, BC PNSD was calculated using average refractory particle number fraction (as a proxy for BC), and PNSD measured in stationary measurement container in Quezon city, Metro Manila. The size-dependent DF was taken from Kecorius et al. [15]. This is because of the current limitation of MERDOC measurement system (i.e., PNSD of BC was not possible to determine, limiting the possibility to model DF using e.g., MPPD). The MV was taken from Natera et al. [16]. The calculated DDR (referred to as DDR*_in silico_*; calculated using Eq. S2) represents the case when the majority of required parameters (DF, MV, BC PNSD) to calculate DDR is taken from literature, and although more advanced than subsequently used method, does not necessarily represent real-world situation. Secondly, DDR was calculated assuming DF = 1 (following Eq. S1). In this instance, oversimplified DDR (referred as DDR*_ab initio_*) assumes that all inhaled particles are deposited in respiratory tract. Although being unreasonable (for urban aerosol), this assumption is often used in scientific literature to calculate the potential deposition dose of airborne pollutants [17–19].

The calculation of the DDR*_ab initio_* of BC and the DDR*_in silico_* of refractory particles was based on the procedure described in Madueño et al. [20] and Kecorius et al. [15], respectively. The following mathematical formulation was adopted:

${DDR}_{ab initio}=MV\times{DF}_{ab initio}\times C_{exp}$ (S1)

${DDR}_{in silico} =MV\times\rho\int_{Dp1}^{Dp2} {DF}_{model} \times PVSD\times dlogDp$ (S2)

where MV is the minute ventilation (L min^-1^), C_exp_ is the BC exposure concentration, $\rho$ is assumed soot density [21] of 1.8 g cm^-3^, used to convert between particle number and mass size distribution, D_p_ is a volume equivalent particle diameter (nm), the DF_ab initio_ is the deposition fraction equal to 1, which means 100% of inhaled particles are deposited in the respiratory system while the DF*_model_* is the deposition fraction taken from Kecorius et al. [15] (average DF from different studies), and PVSD is the particle volume size distribution (μm cm^-3^). The refractory particle number size distribution was reconstructed by multiplying the ambient particle number size distribution (PNSD, measured using mobility particle size spectrometer) with the number fraction of externally mixed particles in a measured PNSD size range. The volume-equivalent diameter was obtained using an empirical, size-dependent aerodynamic shape factor [21].

Table S3. Mean DDR estimated using different assessment methods.

| Assessment Method | DF, % | MV, L min^-1^ | DDR of BC, μg h^-1^ |  |
| --- | --- | --- | --- | --- |
| *Ab initio*  Stationary  Mobile | 100  100 | 23.3*  23.3* | 38.5  68.4 |  |
| *In Silico*  Stationary | Kecorius et al. [15] | 23.3* | 7.4** |  |
| *In Situ (measured)*  Mobile | 43 | 7.8 | 13.9 |  |
| *Based on Natera *et al*.[16]; **Refractory particles, proxy as BC; | | | | |

The results show that by taking the breathing parameters from the anatomical report (intended for the Filipino population), the calculated DDR is up to 3 times higher (due to 3 times higher MV). Similarly, the DDR of BC would be underestimated (approx. by 2-fold) when using MV from the U.S EPA handbook. An overestimation further occurs (of approx. 2 times) if DF is assumed to be 1. In the case of DDR*_ab initio_*, the overall overestimation of DDR due to incorrect MV and DF is from 3 to 5 times (compared to experimentally determined DDR). Contrary, the DDR calculated by using not only literature values of breathing parameters but also assumed DF and BC PNSD (referred to as DDR*_in silico_*) showed to be approx. 2 times lower compared to experimentally determined values. The underestimation occurs even though the considered MV is 3 times higher than the measured value. This further highlight not only the importance of the subject-specific breathing rates but also true PNSD when calculating RTD. If previously mentioned methods, used to estimate DDR, would be applied in health assessment studies, this would yield either diminished (in case of overestimating DDR of BC) or exaggerated (in case of underestimating DDR of BC) effect of BC pollution onto personal health.


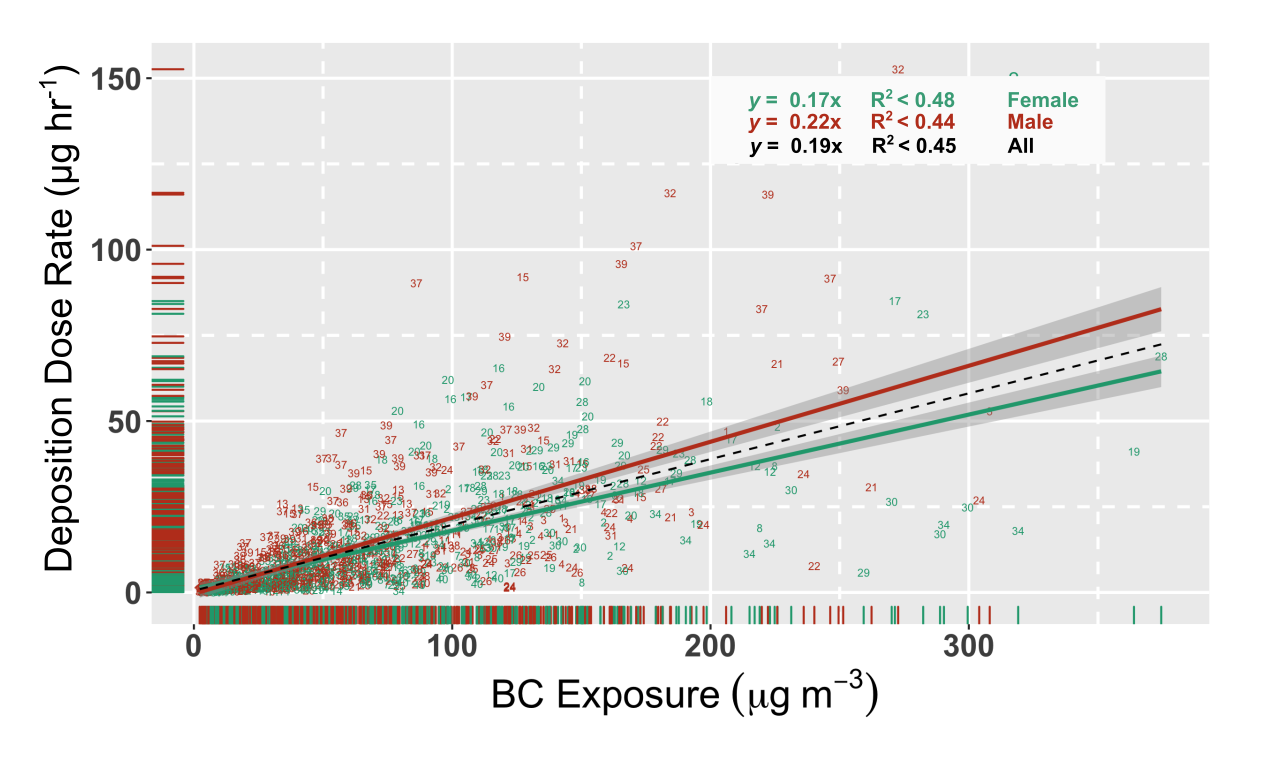


Figure S7. Deposition dose rate as a function of measured BC exposure concentrations. Each volunteer was given a number code, the color represents the male (orange), female (green), and both genders (black). The grey shaded area shows a 95% confidence interval. Lines show the line of best fit.

**References:**

1. Daigle CC, Chalupa DC, Gibb FR, Morrow PE, Oberdörster G, Utell MJ, et al. Ultrafine particle deposition in humans during rest and exercise. Inhal Toxicol. 2003;15:539–52.

2. Chalupa DC, Morrow PE, Oberdörster G, Utell MJ, Frampton MW. Ultrafine particle deposition in subjects with asthma. Environ Health Perspect. 2004;112:879–82.

3. Morawska L, Hofmann W, Hitchins-Loveday J, Swanson C, Mengersen K. Experimental study of the deposition of combustion aerosols in the human respiratory tract. J Aerosol Sci. 2005;36:939–57.

4. Löndahl J, Pagels J, Swietlicki E, Zhou J, Ketzel M, Massling A, et al. A set-up for field studies of respiratory tract deposition of fine and ultrafine particles in humans. J Aerosol Sci. 2006;37:1152–63.

5. Löndahl J, Massling A, Pagels J, Swietlicki E, Vaclavik E, Loft S. Size-resolved respiratory-tract deposition of fine and ultrafine hydrophobic and hygroscopic aerosol particles during rest and exercise. Inhal Toxicol. 2007;19:109–16.

6. Londahl J, Pagels J, Boman C, Swietlicki E, Massling A, Rissler J, et al. Deposition of biomass combustion aerosol particles in the human respiratory tract. Inhal Toxicol. 2008;20:923–33.

7. Löndahl J, Massling A, Swietlicki E, Bräuner EV, Ketzel M, Pagels J, et al. Experimentally determined human respiratory tract deposition of airborne particles at a busy street. Environ Sci Technol. 2009;43:4659–64.

8. Jakobsson JKF, Hedlund J, Kumlin J, Wollmer P, Löndahl J. A new method for measuring lung deposition efficiency of airborne nanoparticles in a single breath. Sci Rep. Nature Publishing Group; 2016;6:1–10.

9. Rissler J, Nicklasson H, Gudmundsson A, Wollmer P, Swietlicki E, Löndahl J. A set-up for respiratory tract deposition efficiency measurements (15–5000 nm) and first results for a group of children and adults. Aerosol Air Qual Res. 2017;17:1244–55.

10. Lin CW, Huang SH, Chang KN, Kuo YM, Wu HD, Lai CY, et al. Experimental measurements of regional lung deposition in taiwanese. Aerosol Air Qual Res. 2019;19:832–9.

11. Madueño L, Kecorius S, Löndahl J, Müller T, Pfeifer S, Haudek A, et al. A new method to measure real-world respiratory tract deposition of inhaled ambient black carbon. Environ Pollut. 2019;248:295–303.

12. Guo L, Johnson GR, Hofmann W, Wang H, Morawska L. Deposition of ambient ultrafine particles in the respiratory tract of children: A novel experimental method and its application. J Aerosol Sci [Internet]. Elsevier Ltd; 2020;139:105465. Available from: https://doi.org/10.1016/j.jaerosci.2019.105465

13. Rissler J, Swietlicki E, Bengtsson A, Boman C, Pagels J, Sandström T, et al. Experimental determination of deposition of diesel exhaust particles in the human respiratory tract. J Aerosol Sci. 2012;48:18–33.

14. Muala A, Nicklasson H, Boman C, Swietlicki E, Nyström R, Pettersson E, et al. Respiratory Tract Deposition of Inhaled Wood Smoke Particles in Healthy Volunteers. J Aerosol Med Pulm Drug Deliv. 2015;28:237–46.

15. Kecorius S, Madueño L, Löndahl J, Vallar E, Galvez MC, Idolor LF, et al. Respiratory tract deposition of inhaled roadside ultrafine refractory particles in a polluted megacity of South-East Asia. Sci Total Environ [Internet]. Elsevier B.V.; 2019;663:265–74. Available from: https://doi.org/10.1016/j.scitotenv.2019.01.338

16. Natera ES, Cuevas CD, Azanon EM, Palattao MB, Espiritu RT, Cobar MC, et al. Compilation of Anatomical, Physiological and Dietary Characteristics for a Filipino Reference Man. 1998; Available from: https://inis.iaea.org/search/search.aspx?orig_q=RN:29028124

17. Dons E, Int Panis L, Van Poppel M, Theunis J, Wets G. Personal exposure to Black Carbon in transport microenvironments. Atmos Environ [Internet]. Elsevier Ltd; 2012;55:392–8. Available from: http://dx.doi.org/10.1016/j.atmosenv.2012.03.020

18. Morales Betancourt R, Galvis B, Balachandran S, Ramos-Bonilla JP, Sarmiento OL, Gallo-Murcia SM, et al. Exposure to fine particulate, black carbon, and particle number concentration in transportation microenvironments. Atmos Environ [Internet]. Elsevier Ltd; 2017;157:135–45. Available from: http://dx.doi.org/10.1016/j.atmosenv.2017.03.006

19. Merritt AS, Georgellis A, Andersson N, Bero Bedada G, Bellander T, Johansson C. Personal exposure to black carbon in Stockholm, using different intra-urban transport modes. Sci Total Environ [Internet]. The Authors; 2019;674:279–87. Available from: https://doi.org/10.1016/j.scitotenv.2019.04.100

20. Madueño L, Kecorius S, Andrade M, Wiedensohler A. Exposure and respiratory tract deposition dose of equivalent black carbon in high altitudes. Atmosphere (Basel). 2020;11:1–14.

21. Park K, Kittelson DB, Zachariah MR, Mcmurry PH. Measurement of inherent material density of nanoparticle agglomerates. 2004;267–72.
